# Anti-Inflammatory Natural Products

**DOI:** 10.1155/2015/608613

**Published:** 2015-04-27

**Authors:** Yifu Yang, Lifei Hou, Abdelfattah El Ouaamari, Lijun Xin

**Affiliations:** ^1^Laboratory of Immunology and Virology, Shanghai University of Traditional Chinese Medicine, Shanghai 201203, China; ^2^Program in Cellular and Molecular Medicine, Boston Children's Hospital and Department of Pediatrics, Harvard Medical School, Boston, MA 02115, USA; ^3^Joslin Diabetes Center, Harvard Medical School, Boston, MA 02115, USA; ^4^Division of Infectious Diseases, Cincinnati Children's Hospital Medical Center, Cincinnati, OH 45229, USA

Inflammation is the first biological response of the immune system against infection or irritation. However, accumulating epidemiological and clinical study indicates that zealous acute inflammation or chronic inflammatory reaction is a significant risk factor to develop various human diseases. Controlling or modulating inflammation is therefore important to prevent or ameliorate certain diseases, such as organ transplantations, allergic diseases, and autoimmune diseases.

Natural products have played an important role throughout the world in treating and preventing human diseases for thousands of years, and, over the past few decades, great efforts have been made to explore modern preparations of natural products with higher efficacy and lower toxicity. Indeed, it is particularly impressive that most of the immunosuppressants are initially derived from natural products including mycophenolic acid (MPA), cyclosporin A (CsA), rapamycin, tacrolimus (FK506), and fingolimod (FTY720) (summarized in review [1]). In addition, several clinical trials carried out in the USA have already shown significant benefits of* T. wilfordii* extract in patients with rheumatoid arthritis (summarized in review [2]). Moreover, recent advances in chemistry and biology have introduced new technologies to synthesize or purify components from natural products and also improved the studies of the underlying mechanisms of action.

This special issue will introduce you to the valuable research reports on anti-inflammatory natural products, ranging from basic researches to exploring roles of natural products against inflammatory diseases. We hope this timely special issue will encourage the research and development of valuable natural products and finally lead to the development of novel therapeutic agents to provide better care to patients.

*Yifu Yang* *Yifu Yang* *Lifei Hou* *Lifei Hou* *Abdelfattah El Ouaamari* *Abdelfattah El Ouaamari* *Lijun Xin* *Lijun Xin*
